# Recyclable photocatalyst perovskite as a single-electron redox mediator for visible-light-driven photocatalysis gram-scale synthesis of 3,4-dihydropyrimidin-2-(1*H*)-ones/thiones in air atmosphere

**DOI:** 10.1038/s41598-023-37526-x

**Published:** 2023-06-24

**Authors:** Farzaneh Mohamadpour

**Affiliations:** School of Engineering, Apadana Institute of Higher Education, Shiraz, Iran

**Keywords:** Chemistry, Catalysis, Photocatalysis

## Abstract

Based on the Biginelli reaction of β-ketoesters, arylaldehydes, and urea/thiourea, we created a green radical synthesis procedure for 3,4-dihydropyrimidin-2-(1*H*)-ones/thiones. A single-electron redox mediator was applied to a solution of ethanol in an air environment, at room temperature, and with blue LEDs as a renewable energy source in order to create. The objective of this research is to create a halide perovskite that is widely available, affordable, recyclable, and economically feasible. A factor mentioned in the discussion is that the procedure tolerates a variety of donating and withdrawing functional groups while still offering a very fast rate and excellent yields. The range of yields is quite uniform (86–94%, average: 90.4%), and the range of reaction times is very quick (4–8 min, average: 5.8 min). Furthermore, gram-scale cyclization shows that it is applicable for use in industry. Additionally, CsPbBr_3_ is quite stable and can be used six times in a row without experiencing significant structural changes or activity loss, which has been extremely helpful in meeting industrial needs and environmental issues.

## Introduction

Perovskites have emerged as one of the foremost optoelectronic materials in recent times on account of their notable characteristics such as extended carrier lifetime, proficient band gap adjustability, exceptional absorption coefficient, robust nonlinear response, elevated electron/hole mobility, as well as efficient processing methodologies. Perovskite materials have been extensively utilized in various domains, including but not limited to light-emitting diodes, photodetectors, laser transmitters, electrochemical reactions, and photoanodes employed in dye-sensitized solar cells^1–7^. As per the findings of^8^, the perovskite-based solar cells have achieved a pinnacle level of photoelectric conversion efficiency, measuring up to 25.2%. Since 2018, CsPbBr_3_ perovskites have garnered interest as a novel type of heterogeneous photocatalyst for certain photocatalytic organic synthesis processes^9^. This is attributed to their notable photovoltaic properties, simplistic operation, and recyclability. Despite the advancements made in recent years, the utilization of perovskites as a form of heterogeneous photocatalysts in the field of organic chemistry has remained in its preliminary stages. This is evidenced by the limited range of reaction types that can currently be achieved^10,11^.

**Figure 1:**
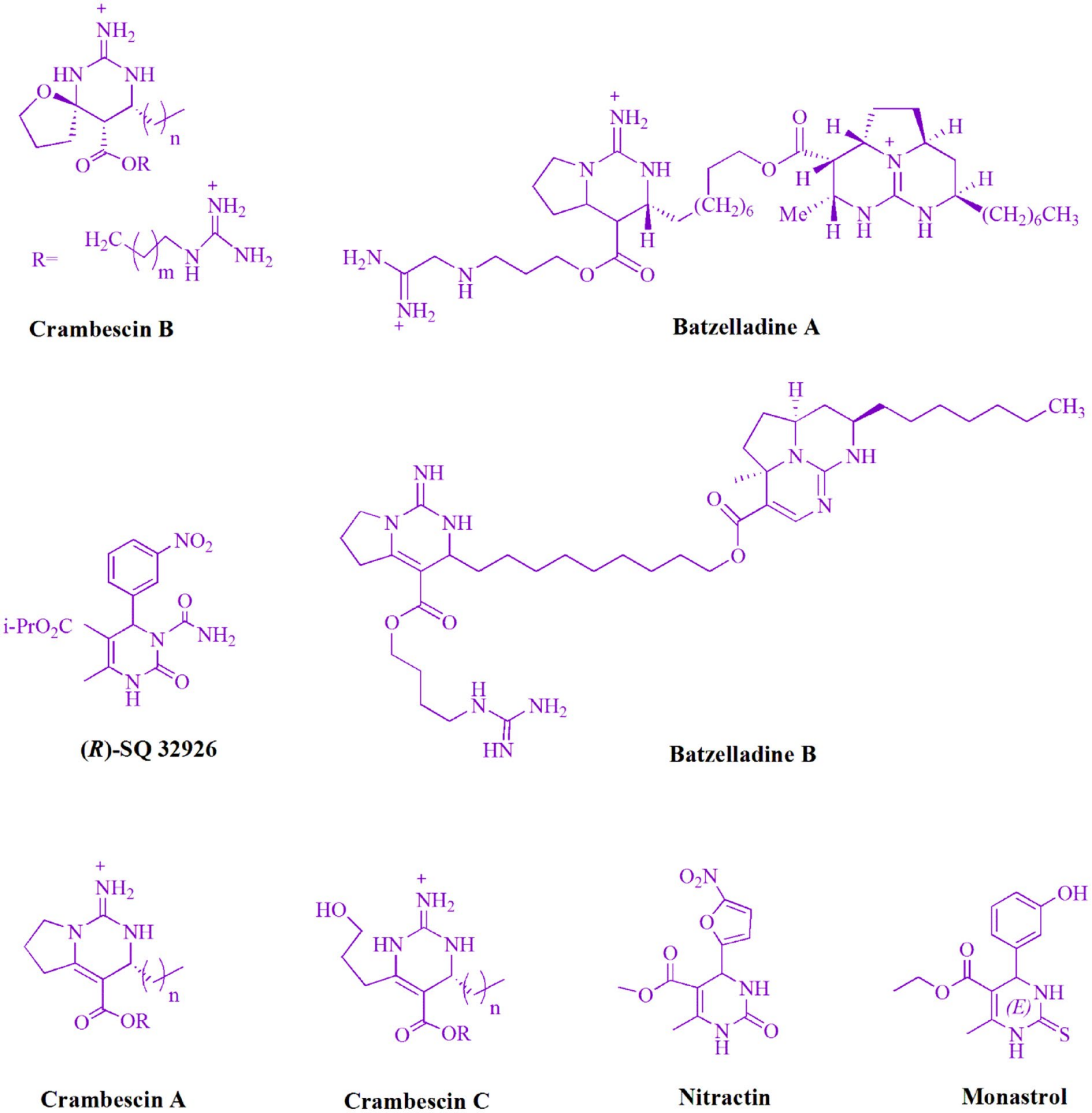
The dihydropyrimidine motifs with pharmaceutical activity.

The dihydropyrimidine structure is believed to have biological and pharmacological attractions (Fig. 1): Calcium channel blockers, antihypertensive effects^12^, anticancer^13^, anti-HIV agent^14^, antibacterial and antifungal^15^, antiviral^16^, antioxidative^17^, and anti-inflammatory^18^.

A multitude of catalysts may be implemented in the synthetic production of 3,4-dihydropyrimidin-2-(1*H*)-ones/thiones, including Cu/Cu_2_O@g-C_3_N_4_^19^, *N*-(phenylsulfonyl)benzene sulfonamide^20^, *h*-BN/Fe_3_O_4_/Co^21^, Na_2_ eosin Y^22^, copper(II)sulfamate^23^, bakers, yeast^24^, hydrotalcite^25^, hexaaquaaluminium (III) tetrafluoroborate^26^, TBAB^27^, copper (II) tetrafluoroborate^28^, [Btto][*p*-TSA]^29^, triethylammonium acetate^30^, saccharin^31^, caffeine^32^, zirconium(IV)-salophen perfluorooctanesulfonate^33^, H_3_[PW_12_O_40_]^34^, Dioxane-HCl^35^, 4CzIPN^36^, H_4_[W_12_SiO_40_]^37^, Zr(H_2_PO_4_)_2_^38^, GO-chitosan^39^, and sodium dodecyl sulfate^40^. The prolonged reaction times, the exorbitant cost of reagents, tumultuous reaction mechanisms, and minimal product yields, foray into waste management concerns. Furthermore, the extraction of homogeneous catalysts from reaction mixtures poses a challenging task. In recent times, photocatalysts have been employed for the purpose of synthesizing heterocyclic chemicals^41–43^. It is noteworthy to highlight that the majority of widely used photocatalysts are essentially homogeneous in nature and consequently, they cannot be efficiently recovered in established techniques. Furthermore, the practical applications of these materials have been constrained by their high costs, elaborate synthetic preparations, requisite air-free reaction conditions, and often limited activity. This has led to their limited use in various fields. Consequently the advancement of heterogeneous photocatalysts that are facile to be fabricated and possess the attribute of facile catalytic separation and reusability is currently in high demand and thus holds significant importance. Over the past few years, there has been considerable interest in perovskites as a viable material for the efficient conversion of solar energy. This is attributed to several noteworthy advantages including their structural simplicity, exceptional optoelectronic properties, flexibility, and robust photo/thermal stability. It is noteworthy that these materials are frequently employed in a broad array of applications, including solar cells, photodetectors, light-emitting diodes (LEDs), semiconductor lasers, and field-effect transistors. Nevertheless, the utilization of perovskites as photocatalysts in inducing organic bond formation remains relatively nascent, with much room for further exploration and development. In light of the optimum band gap of CsPbBr_3_ for the absorption of visible light and the redox potential that grants the ability to function as a solitary-electron redox mediator, a variety of sophisticated organic reactions catalyzed by CsPbBr_3_ have been documented. There remains a strong desire for additional analysis and investigation of innovative organic transformations facilitated by halide perovskites acting as photocatalysts, in order to produce high-value-added compounds via photosynthesis^44^. This discourse expresses our unwavering concern for the evolution of innovative sustainable synthetic techniques. In this regard, our current work expounds on our recent achievement; a visible-light-promoted procedure, which facilitated the successful undertaking of the Biginelli reaction of arylaldehydes in the presence of urea/thiourea and β-ketoesters employing reusable CsPbBr_3_ as a heterogeneous photocatalyst. A noteworthy observation is a facile recovery and the ability to reutilize the photocatalyst for a minimum of six iterations while maintaining its aptitude for catalytic activities.

The current study utilizes a three-condensation domino reaction that exploits a single-electron transfer (SET) pathway. The system also takes advantage of blue light as a renewable energy source and air conditions, while operating at room temperature using ethanol as an environmentally benign solvent. The implementation of this particular approach offers a multitude of significant benefits, notably including superior step economy, expansive substrate scope, the ability to recycle the employed catalyst, and the utilization of eco-friendly solvents. Despite its successful completion devoid of any complications, punctually and within the financial parameters.

## Experimental

### General

The melting points of each chemical were determined utilizing a 9100 electrothermal instrument. Furthermore, the ^1^HNMR and ^13^CNMR spectra were obtained utilizing DMSO-d_6_ in conjunction with the Bruker DRX-300, DRX-400, and DRX-100 Avance instruments. Significant quantities of reagents were supplied by Fluka, Merck, and Acros, and expeditiously employed for the purposes intended.

#### A methodology for generating 3,4-dihydropyrimidin-2-(1H)-ones/thiones (4a-w)

In the presence of a 1 mol% loading of CsPbBr_3_, urea/thiourea (**2**, 1.5 mmol), ethyl/methyl acetoacetate (**3**, 1.0 mmol), and arylaldehyde derivatives (**1**, 1.0 mmol) were stirred at room temperature using 3 mL of EtOH as a solvent. The data was collected and documented via Thin-Layer Chromatography (TLC). Following the reaction, the resultant mixture underwent a thorough screening and washing protocol with EtOH. Subsequently, the crude solid was subject to crystallization using ethanol as a solvent, thereby obtaining the pure and pristine material without any supplementary purification measures. We aim to investigate the feasibility of synthesizing the aforementioned chemicals on a gram-scale within the context of pharmaceutical process research and development (R&D). In a single experimental procedure, 50 mmol of 4-methylbenzaldehyde, 75 mmol of urea, and 50 mmol of methyl acetoacetate were employed. Following a reaction duration of 4 min, the resultant product was acquired through the implementation of standard filtration methodology. Based on the ^1^HNMR spectrum, it can be inferred that the chemical is spectroscopically pure. Through the analysis of the spectral characteristics of the products, specifically in terms of the ^1^HNMR data, we have categorized them accordingly. The aforementioned Supporting Information document provides an extensive compilation of pertinent information.

## Results and discussion

**Figure 2:**
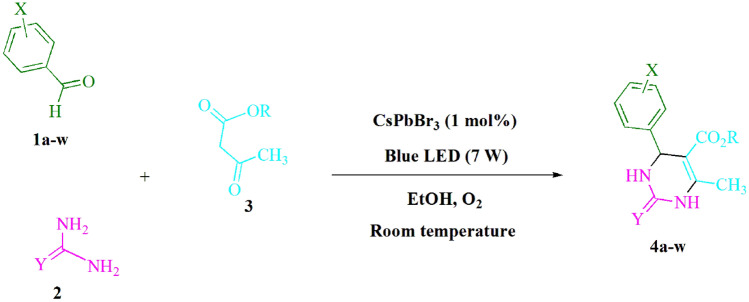
The present study proposes a methodology for synthesizing 3,4-dihydropyrimidin-2-(1*H*)-ones/thiones.

The present study aimed to investigate the interactions among benzaldehyde (1.0 mmol), urea (1.5 mmol), and ethyl acetoacetate (1.0 mmol) in EtOH solvent (3 mL). Upon incubation of 3 mL of EtOH without the presence of a photocatalyst for a duration of 30 min, an insignificant level of **4f** was produced at ambient temperature, as illustrated in Table 1, entry 4. The incorporation of multiple supplementary photocatalysts expedited the chemical reaction. The aforementioned substances were observed to include CsPbBr_3_, PbBr_2_, CsBr, methylammonium lead tribromide (MAPbBr_3_), methylammonium lead triiodide (MAPbI_3_), and tricesium nonabromodibismuthate (III) (Cs_3_Bi_2_Br_9_). Through the application of this technique, the synthesis of **4f** can be accomplished with a varying yield between 32 and 94% as documented in Table 1. The aforementioned results facilitated the enhanced efficacy of CsPbBr_3_. According to the data presented in Table 1 in entry 2, the implementation of 1 mol% CsPbBr_3_ led to a production yield of 93%. Table 2 at entry 10, shows that under solvent-free conditions, only 42% of product **4f** is produced. Table 2 shows that the outcomes for toluene, CH_2_Cl_2_, DMSO, EtOAc, ethyl lactate, THF, MeOH, CH_3_CN, and DMF were noticeably lower; whereas the employment of EtOH as green solvent caused an increase in yield and facilitated the process. The reaction in ethanol demonstrated notable levels of both yield and reaction rate. Table 2, entry 1 provided the necessary data from which a yield of 93% was realized. Various light sources have been investigated in numerous studies to determine the impact of blue light on yield (as depicted in Table 2). The detection of **4f** in the trace was facilitated by controlling the test in the absence of the light source. As per the findings of the research, the synthesis of product **4f** necessitates the utilization of CsPbBr_3_ in conjunction with visible light. The optimal parameters were determined via the utilization of blue LED power intensities amounting to 3 W, 7 W, and 10 W. Based on the findings presented in Table 2, entry 1, it can be concluded that the utilization of blue LEDs (7 W) resulted in optimal outcomes. Tests were performed on several substrates, as presented in Table 3 and Fig. 2, under optimal conditions. The resultant effect of the reaction remained unaltered by the presence of the benzaldehyde substituent, as indicated in Table 3. Within the scope of this reaction, substitutions involving polar and halide species were demonstrated to be permissible. The present state of the reaction permits both reactions involving electron-donating functional groups and those involving electron-withdrawing functional groups. The potential yield of aromatic aldehydes substituted in *ortho*, *meta*, or *para* positions is considerably high. Ethyl acetoacetate and methyl acetoacetate exhibit analogous reaction characteristics. The reactiveness of urea and thiourea exhibited comparability.

**Table 1:** A photocatalyst optimization table is made available to facilitate the production of **4f** in an effective manner.


Entry	Photocatalyst	Solvent (3 mL)	Time (min)	Isolated yields (%)
1	CsPbBr_3_ (0.5 mol%)	EtOH	5	81
2	CsPbBr_3_ (1 mol%)	EtOH	5	93
3	CsPbBr_3_ (1.5 mol%)	EtOH	5	94
4	–	EtOH	30	Trace
5	PbBr_2_ (1 mol%)	EtOH	5	65
6	CsBr (1 mol%)	EtOH	5	32
7	MAPbBr_3_ (1 mol%)	EtOH	5	45
8	MAPbI_3_ (1 mol%)	EtOH	5	39
9	Cs_3_Bi_2_Br_9_ (1 mol%)	EtOH	5	57

Reaction conditions: in the experimental protocol, a mixture consisting of benzaldehyde (1.0 mmol), ethyl acetoacetate (1.0 mmol), and urea (1.5 mmol), was subjected to photoreaction utilizing multiple photocatalysts.

**Table 2:** A tabular representation is presented herein elucidating the optimization of both solvent and visible light in the synthesis of **4f.**


Entry	Light source	Solvent (3 mL)	Time (min)	Isolated yields (%)
1	Blue light (7 W)	EtOH	5	93
2	Green light (7 W)	EtOH	5	87
3	White light (7 W)	EtOH	5	85
4	–	EtOH	30	Trace
5	Blue light (3 W)	EtOH	5	86
6	Blue light (10 W)	EtOH	5	93
7	Blue light (7 W)	Toluene	30	18
8	Blue light (7 W)	CH_2_Cl_2_	20	34
9	Blue light (7 W)	DMSO	30	25
10	Blue light (7 W)	–	7	42
11	Blue light (7 W)	EtOAc	5	64
12	Blue light (7 W)	Ethyl lactate	5	47
13	Blue light (7 W)	THF	25	31
14	Blue light (7 W)	MeOH	5	58
15	Blue light (7 W)	CH_3_CN	5	76
16	Blue light (7 W)	DMF	25	28

Reaction conditions: in the experimental procedure, a quantity of CsPbBr_3_ (1 mol%) coalesced with 1.0 mmol of benzaldehyde, 1.0 mmol of ethyl acetoacetate, and 1.5 mmol of urea.

**Table 3:** The present study involves the production of 3,4-dihydropyrimidin-2-(1*H*)-ones/thiones, carried out through the use of recyclable halide perovskite as a single-electron redox mediator.


Entry	X	R	Y	Product	Time (min)	Isolated yield %	M.p. °C	Lit. M.p. °C
1	4-Me	C_2_H_5_	O	**4a**	4	92	218–220	216–217^38^
2	4-O_2_N	CH_3_	O	**4b**	5	91	215–217	214–216^26^
3	4-F	CH_3_	S	**4c**	5	93	212–214	210–212^32^
4	3-OMe	C_2_H_5_	O	**4d**	6	94	205–207	205–206^24^
5	4-OMe	C_2_H_5_	O	**4e**	6	91	201–203	202–203^25^
6	H	C_2_H_5_	O	**4f**	5	93	199–201	200–202^26^
7	2-Cl	CH_3_	O	**4g**	7	89	250–252	248–252^23^
8	4-Me	CH_3_	O	**4h**	4	94	222–224	220–222^39^
9	3-O_2_N	C_2_H_5_	O	**4i**	4	94	226–228	225–229^34^
10	2,4-di-OMe	C_2_H_5_	O	**4j**	7	91	208–210	210–211^38^
11	4-F	C_2_H_5_	O	**4k**	4	92	175–176	174–176^27^
12	N,N-di-Me	C_2_H_5_	O	**4l**	6	91	252–254	255–257^25^
13	3-Me	C_2_H_5_	O	**4m**	4	90	202–204	203–206^34^
14	4-Cl	CH_3_	O	**4n**	8	87	207–209	205–206^30^
15	2-Cl	C_2_H_5_	O	**4o**	7	88	222–224	220–223^23^
16	3,4,5-tri-OMe	C_2_H_5_	S	**4p**	8	86	196–198	195–197^32^
17	2,4-di-OMe	C_2_H_5_	S	**4q**	7	88	165–167	163–164^38^
18	3-Cl	C_2_H_5_	O	**4r**	7	86	189–191	191–193^23^
19	4-OMe	C_2_H_5_	S	**4s**	7	89	149–151	150–152^26^
20	4-OH	C_2_H_5_	O	**4t**	8	86	231–233	230–232^27^
21	H	C_2_H_5_	S	**4u**	5	92	209–211	208–210^26^
22	4-OMe	CH_3_	O	**4v**	6	93	186–187	186–189^28^
23	2-O_2_N	CH_3_	O	**4w**	4	90	276–278	274–277^28^

Table 4 furnishes data pertaining to the turnover frequency (TOF) and turnover number (TON). The concept of yield is classified into two types, namely TON (Turnover Number) and TOF (Turnover Frequency), which can be expressed as TON = Yield/Amount of catalyst (mol), TOF (min^-1^) = Yield/Time/Amount of catalyst (mol), respectively. Increased values of TON and TOF lead to enhanced catalyst efficiency by diminishing the amount of catalyst necessary for augmenting yields. The transistor **4f** exhibits a high TON and TOF: 93 and 18.6, respectively. Similarly, the transistor **4u** also displays a high TON: 92 and TOF: 18.4. The study's aims of optimizing yield, reducing reaction time, and minimizing recyclable photocatalyst usage are the focal points.

**Table 4:** In order to determine the turnover number (TON) and turnover frequency (TOF), the ensuing computations were performed.

Entry	Product	TON	TOF	Entry	Product	TON	TOF
1	**4a**	92	23	13	**4m**	90	22.5
2	**4b**	91	18.2	14	**4n**	87	10.8
3	**4c**	93	18.6	15	**4o**	88	12.5
4	**4d**	94	15.6	16	**4p**	86	10.7
5	**4e**	91	15.1	17	**4q**	88	12.5
6	**4f**	93	18.6	18	**4r**	86	12.2
7	**4g**	89	12.7	19	**4s**	89	12.7
8	**4h**	94	23.5	20	**4t**	86	10.7
9	**4i**	94	23.5	21	**4u**	92	18.4
10	**4j**	91	13	22	**4v**	93	15.5
11	**4k**	92	23	23	**4w**	90	22.5
12	**4l**	91	15.1				

**Figure 3:**
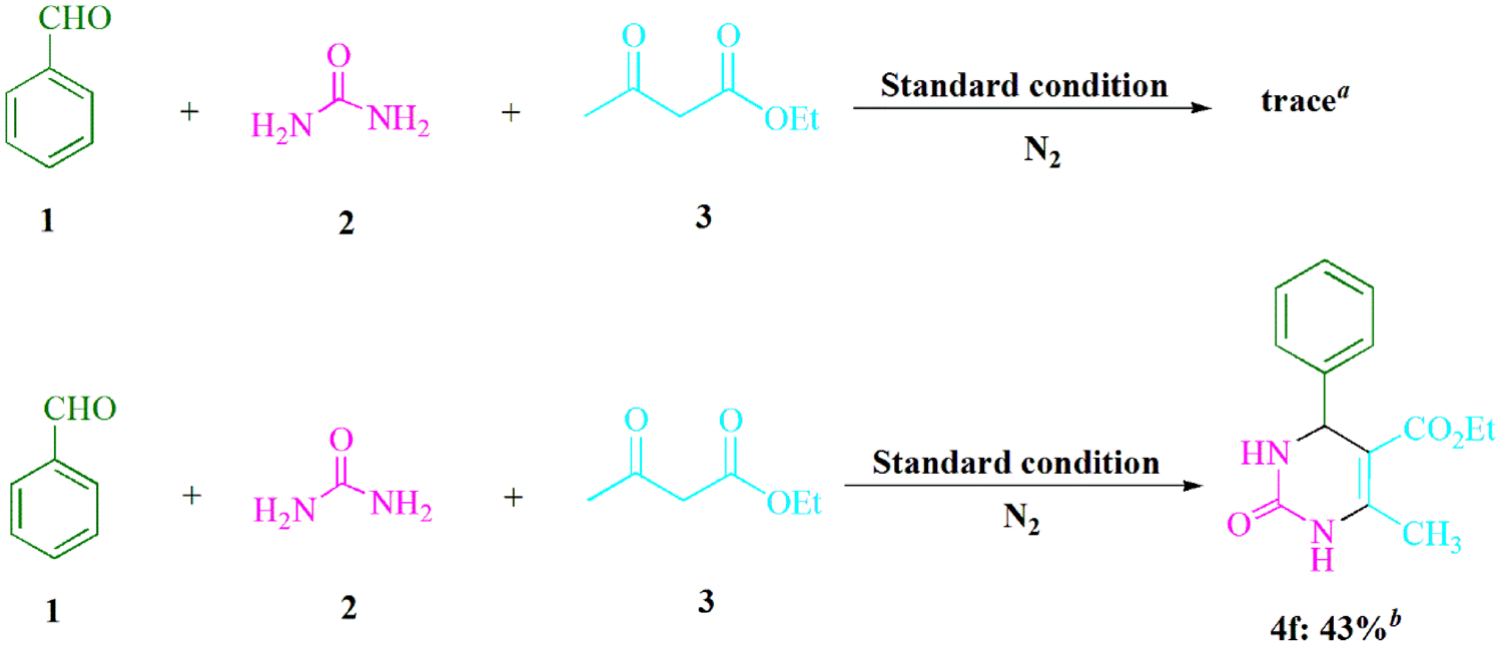
The comprehension of the process of certain reactions is facilitated by conducting significant control studies. For instance, the reactions involving urea (**2**, 1.5 mmol), ethyl acetoacetate (**3**, 1.0 mmol), and benzaldehyde (**1**, 1.0 mmol) are considered pivotal in this regard. ^*a*^The process of removing gas from a reaction through degassing by utilizing the *Freeze–Pump–Thaw* approach; ^*b*^The reaction was purged with N_2_.

In order to assess the significance of air atmosphere, additional controlled experiments were conducted (Fig. 3). Upon purging the reaction with nitrogen gas (N_2_), a yield of 43% for the formation of product **4f** was observed. Nonetheless, the response carried out under stringent anaerobic circumstances using the *Freeze–Pump–Thaw* approach exclusively yielded **4f** in a negligible amount. The reaction was executed under a nitrogen atmosphere that had undergone degassing through the utilization of the *freeze–pump–thaw* method. It was observed that the reaction came to a complete halt, which implied that oxygen (O_2_) played an indispensable role. Nevertheless, in the absence of oxygen (O_2_) during the reaction, it can be postulated that the reaction pathway involving oxygen is seemingly fundamental given the markedly low yield recorded in the oxygen-free control experiment.

**Figure 4:**
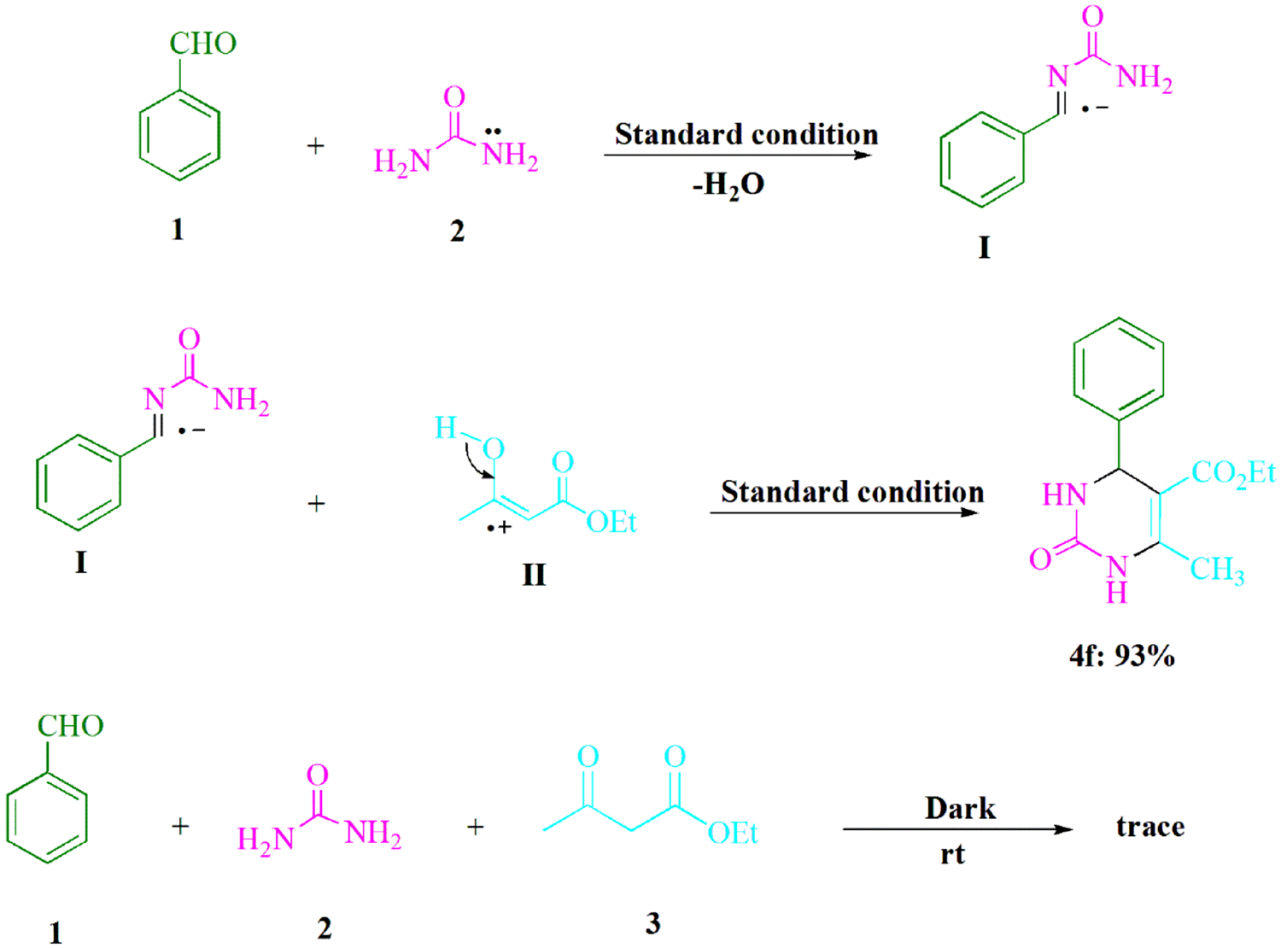
Essential control investigations are furnished by the reactions of urea (**2**, 1.5 mmol), ethyl acetoacetate (**3**, 1.0 mmol), and benzaldehyde (**1**, 1.0 mmol) to facilitate an understanding of their mechanisms.

**Figure 5:**
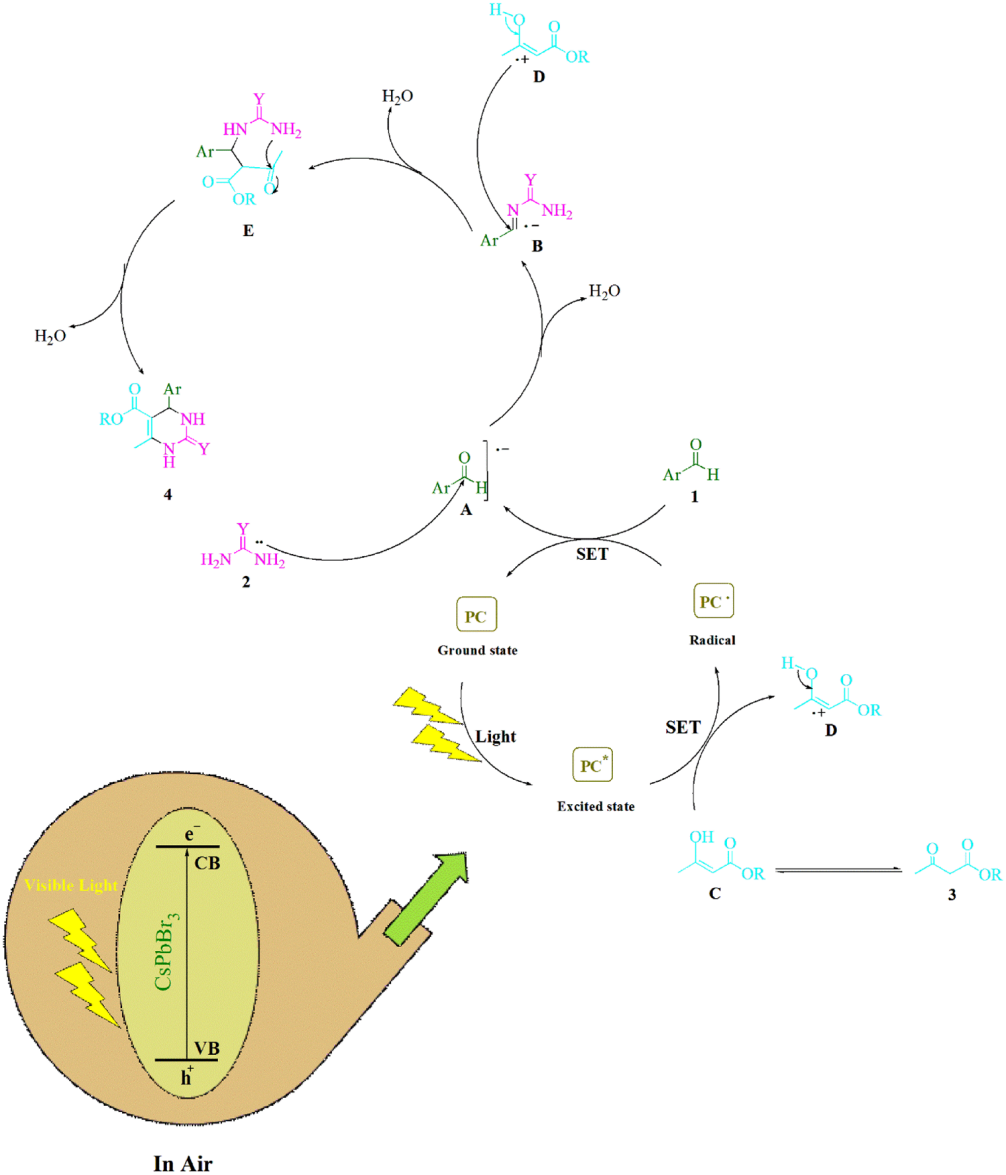
Elaborate details on the synthesis of 3,4-dihydropyrimidin-2-(1*H*)-ones/thiones were provided, wherein the proposed mechanism was explicated. PC: photocatalyst (CsPbBr_3_).

Furthermore, Fig. 4 displays the findings of control examinations conducted to reveal the mechanism that underlies the three-component visible light-induced reaction. The Biginelli reaction is commonly believed to proceed via a two-step mechanism; the initial step involves the generation of benzylideneurea (**I**), whereas the terminal step involves the condensation of (**I**) with ethyl acetoacetate (**3**). The synthesis of benzylideneurea (**I**) was performed via the condensation reaction between benzaldehyde (**1**) and urea (**2**) under conventional conditions, utilizing CsPbBr_3_ as a photocatalyst in ethanol solvent, facilitated by blue light emitting diode irradiation, along with the removal of water molecules. Under conventional conditions, the intended product **4f** was produced in 93% of the interactions occurring between the iminium intermediate (**I**) and cation radical (**II**). Even in the presence of darkness, a discernible presence of product **4f** was generated during the course of the reaction. According to the results of this experiment, Fig. 5 presents a plausible reaction pathway.

### Reaction mechanism

Figure 5 delineates the proposed mechanism with comprehensive elaboration. Upon exposure to visible light, the CsPbBr_3_ underwent the creation of electrons in the conduction band (CB) and holes in the valence band (VB). As a result of being exposed to visible light, the absorption of a photon within CsPbBr_3_ results in the creation of an electron and a hole, denoted as (e^-^) and (h^+^), respectively. These entities are capable of functioning as singular electron donors and acceptors within a variety of desired organic transformations, according to the literature^45^.

The utilization of the single-electron transfer (SET) technique has facilitated the development of visible-light-driven photocatalytic devices employing CsPbBr_3_, which exhibit the capability for recycling and serve as a promising candidate for halide perovskite-based applications. The acceleration of the process is facilitated by the presence of visible light. The present investigation concerns the SET activity of the CsPbBr_3_ radical and arylaldehydes (**1**), which yields the regeneration of the ground-state CsPbBr_3_ and the intermediate (**A**). The formation of a reactive iminium intermediate (**B**) is the consequence of the nucleophilic addition of radical (**A**) to urea/thiourea (**2**). The cation radical (**D**) is generated through the SET method, which entails the utilization of visible light to elevate CsPbBr_3_^*^ to a higher energy state. The present mechanism involves the electrophilic attack of the cation radical (**D**) on the iminium intermediate (**B**), which results in the formation of the cyclized dehydrated product (**4**) as a mechanistic consequence.

### Recyclability of the catalyst

**Figure 6:**
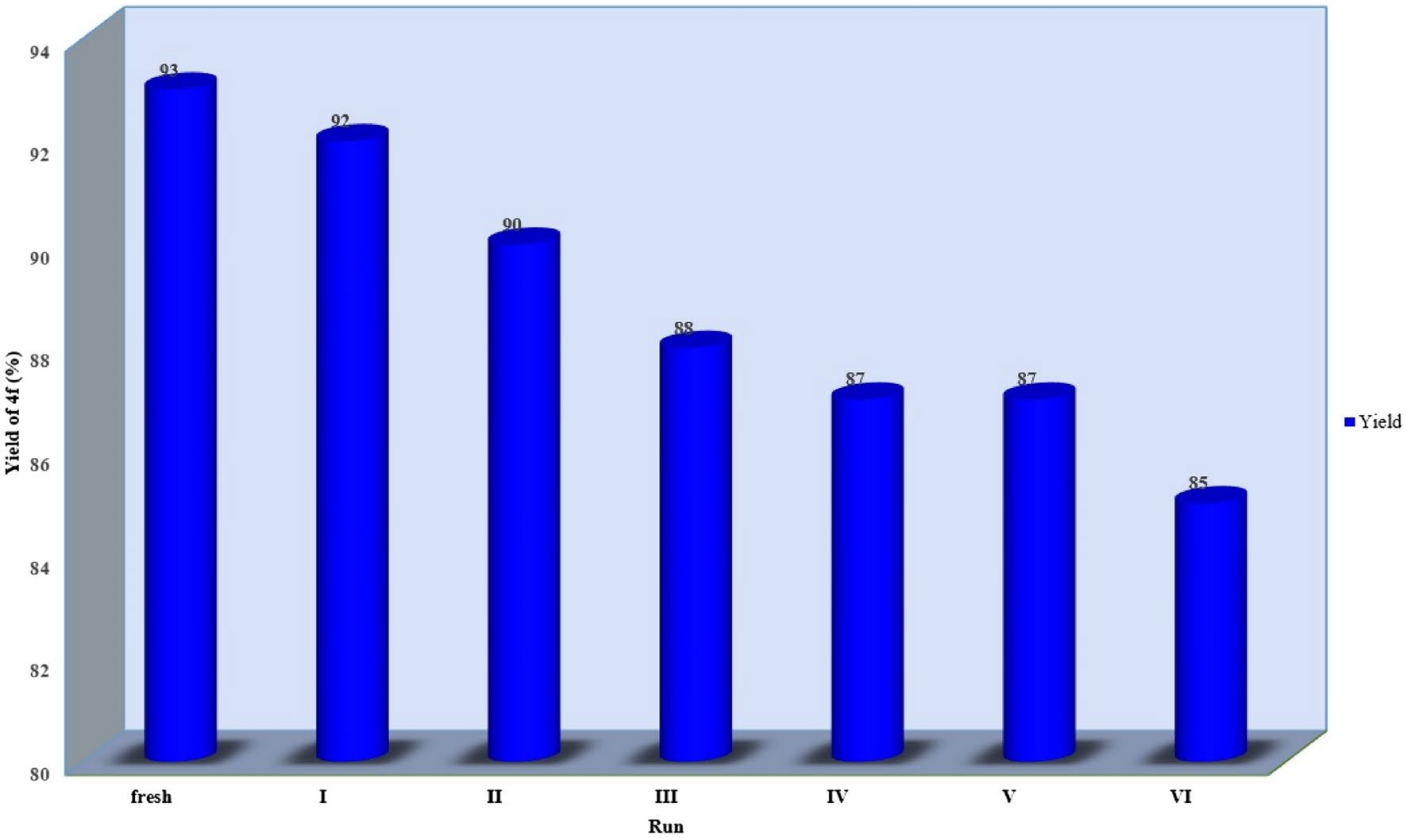
The recyclability potential of CsPbBr_3_ in the synthesis of **4f** is under investigation.

The recycling experiments were conducted to assess the durability and recyclability of CsPbBr_3_. The present study examines the potential for catalyst reusability in relation to CsPbBr_3_ through the synthesis of 5-Ethoxycarbonyl-6-methyl-4-phenyl-3,4-dihydropyrimidin-2(1*H*)-one (**4f**). Upon the culmination of the reaction, the catalyst was removed through the application of centrifugal force, followed by filtration and washing with ethyl acetate (2 × 3 mL). Furthermore, the catalyst was dried using a vacuum without undergoing any additional purification steps prior to its successful reuse in subsequent reaction cycles. The graphical representation is depicted in Fig. 6. The catalyst exhibits a notable degree of reusability with up to six reuse cycles without significant reduction in activity level. This observation is suggestive of the remarkable activity and longevity of the catalyst under study. As an essential component of the work-up protocol, it was possible to salvage the CsPbBr_3_ material and utilize it for the synthesis of **4f** in up to six successive reactions with minimal loss of activity. The obtained results indicated a high level of operational efficiency, as demonstrated by the consistently favorable yield outcomes, namely: fresh (93%), run 1 (92%), run 2 (90%), run 3 (88%), run 4 (87%), run 5 (87%), and run 6 (85%).

Table 5 presents a comparative analysis of the catalytic efficiency of multiple catalysts in the synthesis of 3,4-dihydropyrimidin-2-(1*H*)-ones/thiones. This particular technique may be implemented in environments that are illuminated by visible light, recyclability of catalyst, involve modest quantities of photocatalyst, exhibit rapid reaction kinetics, and are characterized by the lack of any unintended by-products. Atom-economic methodologies exhibit remarkable efficacy and exert a significant influence on the industrial sector on a multigram scale.

**Table 5:** This study investigates the catalytic capacity of numerous catalysts present in the given text toward the synthesis of **4f.**

Entry	Catalyst	Conditions	Time/yield (%)	References
1	Cu/Cu_2_O@g-C_3_N_4_	White LED, EtOH, rt	4.5 h/86	^19^
2	Na_2_ eosin Y	White LED, EtOH, rt	10 min/94	^22^
3	Bakers, yeast	Room temperature	1440 min/84	^24^
4	Hydrotalcite	Solvent-free, 80 °C	35 min/84	^25^
5	[Al(H_2_O)_6_](BF_4_)_3_	MeCN, Reflux	1200 min/81	^26^
6	Cu(BF_4_)_2_.xH_2_O	Room temperature	30 min/90	^28^
7	[Btto][*p*-TSA]	Solvent-free, 90 °C	30 min/96	^29^
8	Triethylammonium acetate	Solvent-free,70 °C	45 min/90	^30^
9	Saccharin	Solvent-free, 80 °C	15 min/88	^31^
10	Caffeine	Solvent-free, 80 °C	25 min/91	^32^
11	4CzIPN	Blue LED, EtOH, rt	5 min/96	^36^
12	CsPbBr_3_	Blue LED, EtOH, rt	5 min/93	This work

The synthesis employs three distinct constituents namely benzaldehyde, ethyl acetoacetate, and urea.

## Conclusion

In the radical Biginelli reaction, 3,4-dihydropyrimidin-2-(1*H*)-ones/thiones were produced by combining aldehydes, β-ketoesters, urea or thiourea. The novel recyclable halide perovskite; CsPbBr_3_ was used to catalyze the reaction using the single-electron transfer (SET) technique of photosynthesis. At room temperature and in an airy setting, blue light can be used to generate a sustainable energy source in an ethanol solution. The method has a number of benefits, such as a quick reaction time, the absence of potentially toxic solvents, high yields, an efficient reaction process, stable conditions, and a renewable energy source. Without altering the outcome, a multigram scale reaction of model substrates can be expedited. Additionally, CsPbBr_3_ was extremely stable and could be reusability for six times without experiencing any substantial structural changes or activity loss. The method can therefore be used in an environment that is both sustainable and profitable.

## Supplementary Information

Supplementary Information.

## Data Availability

All data generated or analyzed during this study are included in this published article [and its supplementary information files].
