# Supplementary material for: Recyclable photocatalyst perovskite as a single-electron redox mediator for visible-light-driven photocatalysis gram-scale synthesis of 3,4-dihydropyrimidin-2-(1H)-ones/thiones in air atmosphere
Source: Sci Rep. 2023 Jun 24;13:10262. doi: 10.1038/s41598-023-37526-x (PMC10290714; doi:10.1038/s41598-023-37526-x)
Supplement: Supplementary file 1 — Supplementary Information. [file 41598_2023_37526_MOESM1_ESM.pdf]

# Supporting Information

# Recyclable photocatalyst perovskite as a single-electron redox mediator for visible-light-driven photocatalysis gram-scale synthesis of 3,4-dihydropyrimidin-2-(1*H*)-ones/thiones in air atmosphere

Farzaneh Mohamadpour *

School of Engineering, Apadana Institute of Higher Education, Shiraz, Iran

* Corresponding author. mohamadpour.f.7@gmail.com

**Contents**

**1. Control experiments**

**1. 1. Control experiments on the synthesis of 4f**

**1. 2. Control experiments on intermediate A**

**1. 3. The effect of daylight and darkness in the reaction mechanism**

**1. 4. The effect of temperature on the reaction**

**1. 5. The effect of visible light on the reaction**

**1. 6. The effect of time on the yield reaction**

**2. Investigating the catalytic activity of a number of photocatalysts**

**3. Single-electron transfer (SET)/photoinduced-electron transfer (PET) pathway**

**4. $^{1}$HNMR and $^{13}$CNMR data for compounds (4a, 4b, 4h, 4j, 4k, 4m, 4o, 4p, and 4u)**

**5. $^{1}$HNMR and $^{13}$CNMR files for compounds (4a, 4b, 4h, 4j, 4k, 4m, 4o, 4p, and 4u)**

**6. References**

## 1. Control experiments

### 1. 1. Control experiments on the synthesis of 4f

Diverse approaches were utilized to obviate intermediates, as explicated in Figures S1 and S2, with the aim of ascertaining the indispensability of a visible light source and photocatalyst for instigating the phase. For the purpose of control, experimental trials pertaining to the intermediate compounds in the absence of a photocatalyst have been carried out. When conducting the procedural steps involving the reaction of benzaldehyde, urea, and ethyl acetoacetate, it was observed that the generated product **4f** was present only in insignificantly small quantities when executed either at room temperature or under the reflux conditions of EtOH in the absence of blue light/$CsPbBr_3$, as indicated in Figure S1: I and II. When subjected to blue light, ethanol, and ambient temperature in the absence of $CsPbBr_3$, a minute amount of the compound **4f** was generated during the reaction between benzaldehyde, urea, and ethyl acetoacetate, as evidenced by Figure S1: III. Moreover, upon conducting the reaction between benzaldehyde, urea, and ethyl acetoacetate in EtOH solution using a $CsPbBr_3$ photocatalyst and under ambient conditions without blue light, a minor quantity of product **4f** was generated (Figure S1: IV).

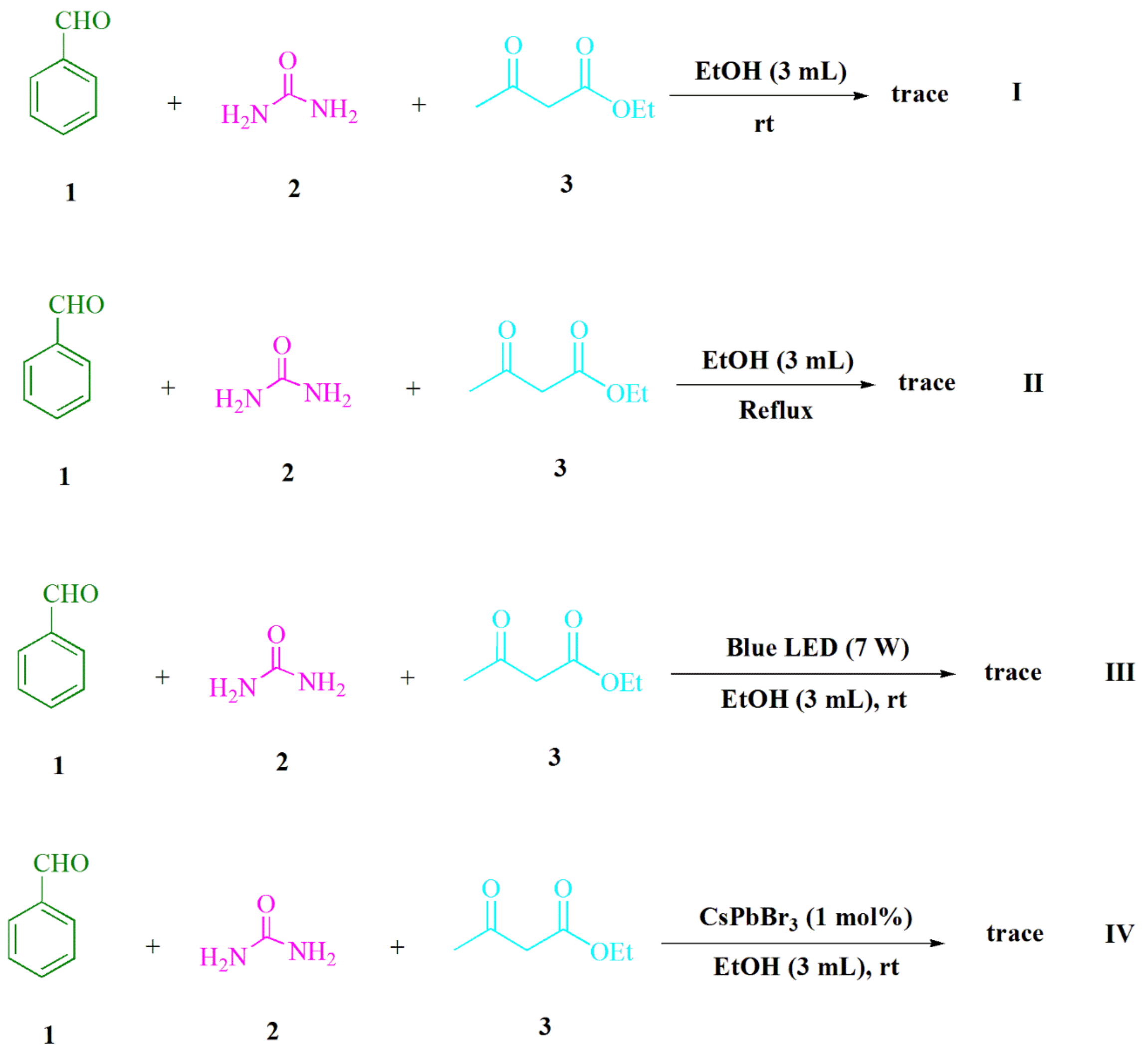


**Figure S1.** Control experiments on the synthesis **4f**.

## 1. 2. Control experiments on intermediate A

To provide additional experimental data for control purposes, trials focusing on the intermediates were performed. The mechanism underlying the Biginelli reaction can be characterized as a process that involves a sequential occurrence of two distinct steps. The initial step involves the synthesis of benzylideneurea (**A**) while the second step entails the condensation of said product

with ketoester (**3**). Benzaldehyde (**1f**) and urea (**2**) were subjected to agitation through visible light exposure using $CsPbBr_3$ and EtOH solution. In accordance with Figure S2: I, the intermediate product designated as (**A**) was identified in 88% of the instances. Furthermore, when (**A**) was subjected to agitation at ambient temperature or refluxed in an ethanol solution containing ethyl acetoacetate (**3**) in the absence of photocatalyst and visible light, a scant quantity of the resulting product **4f** was observed (as shown in Figure S2: II, III). However, the combination of compound (**A**) and ethyl acetoacetate (**3**) in the presence of $CsPbBr_3$ and EtOH in the absence of visible light resulted in the production of a negligible quantity of compound **4f** (as demonstrated in Figure S2: IV. Moreover, following the amalgamation of compound (**A**) and ethyl acetoacetate (**3**) in the existence of visible light and ethanol in the nonattendance of $CsPbBr_3$ photocatalyst, an infinitesimal quantity of **4f** was generated (as depicted in FigureS2: V). The results obtained from the conducted control experiments indicate that the advancement of the reactions under consideration necessitates the presence of $CsPbBr_3$ as well as a blue LED irradiation.

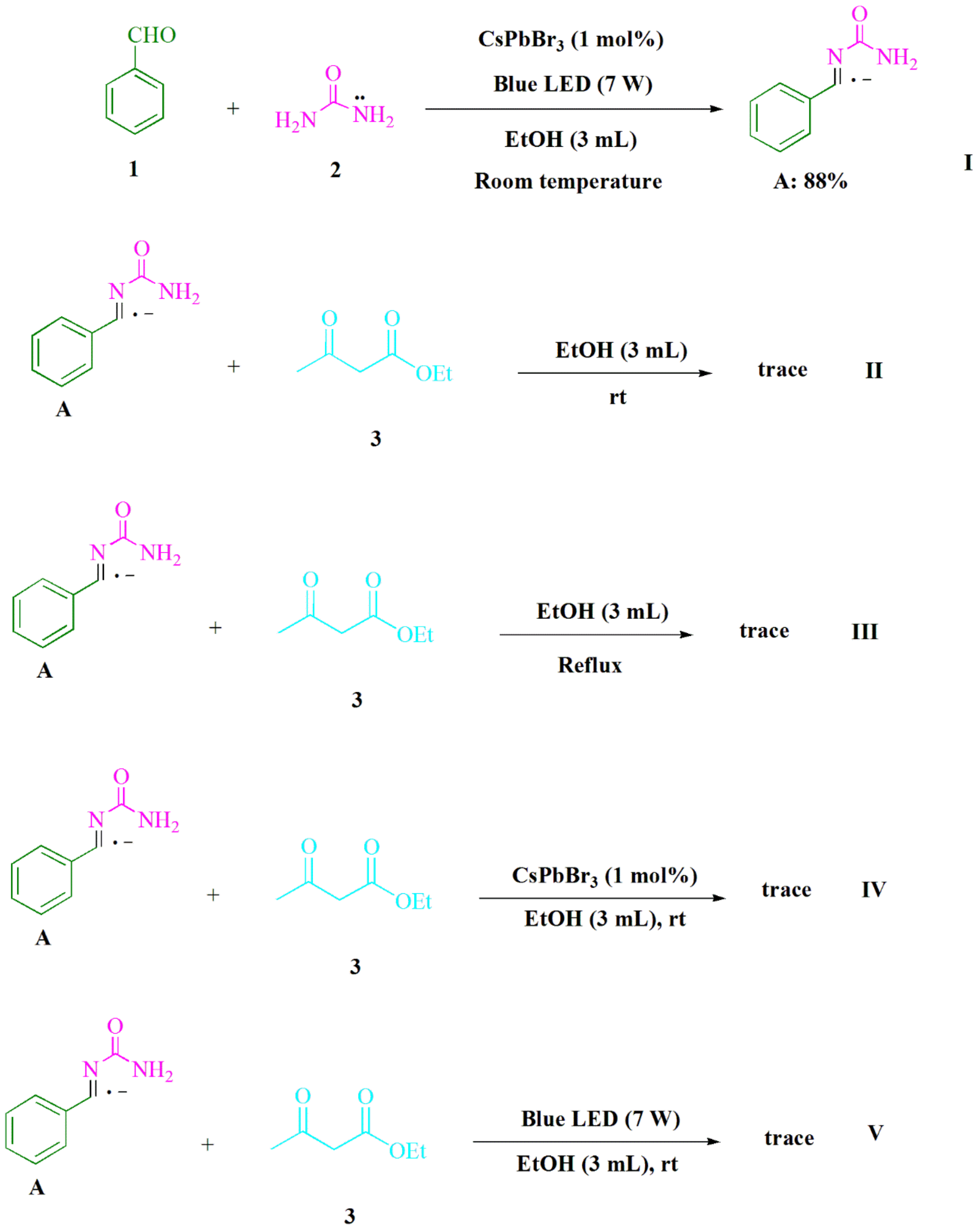


**Figure S2.** Control experiments on intermediate **A**.

## 1. 3. The effect of daylight and darkness in the reaction mechanism

The present study was primarily centered on investigating the interplay between daylight/darkness and visible light in the chemical reaction mechanism. This study endeavors to explore a sequence of reactions for the purpose of evaluating the influence of diurnal fluctuation on the efficacy of EtOH (3 mL) and $CsPbBr_3$ (1 mol%). The observations suggest that the omission of detectable wavelengths of light corresponded with a substantial decrease in the output of the substance.

The yield (%) obtained under the influence of daylight in the synthesis of **4f**: trace

The yield (%) obtained under the influence of darkness in the synthesis of **4f**: trace

**1. 4. The effect of temperature on the reaction**

Several control experiments were carried out utilizing established methods to verify the essentiality of visible light radiation for the examined phase. A research investigation was conducted to evaluate the ramifications of exposure to visible light radiation on the aforementioned procedure. Sustained exposure to light was found to be a necessary requirement for the induction of these reactions. The retardation of reaction kinetics and suboptimal product yields were observed when visible light irradiation was absent. The reality of the situation is that the reactions were initiated via the utilization of visible light radiation. The blue LED, commonly referred to as a light-emitting diode, functions as a significant source of visible light energy that is widely employed across numerous processes, as demonstrated in Figure S3.

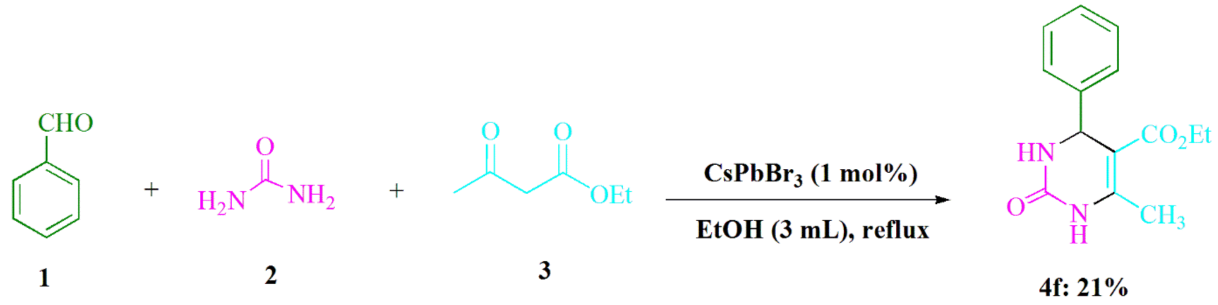

**Figure S3.** The influence of temperature on the reaction.

## 1. 5. The effect of visible light on the reaction

The present study has revealed that the use of a visible light source was deemed essential to achieve significant conversion rates. Standard heating at temperatures of 90-100 °C under normal atmospheric conditions proved inadequate for process. The photoreactivity of visible light coupled with the rapid vibrational motion of chemical bonds facilitates rapid and frequent reactant collisions, ultimately resulting in rapid chemical transformation.

In this reaction, the distance between the visible light source and the reaction vessel was 3.5 cm, and also according to the control experiments that were done, visible light radiation must be continuous for the reaction to progress. In addition, the visible light irradiation is collimated.

The investigation's findings reveal that $CsPbBr_3$ undergoes prompt intersystem crossing from its basal state to its excited state upon photoexcitation. The enhanced rate of chemical reaction may potentially result from the concomitant effect of exposure to visible light radiation and $CsPbBr_3$. Organic photocatalysts, such as $CsPbBr_3$, have demonstrated the potential to function as an alternative to transition metal photo-redox catalysts, in light of their enhanced economic and environmental attributes. Extensive investigations have been conducted to examine the

photochemical behavior of $CsPbBr_3$ in response to visible light excitation. These investigations have concluded that rapid intersystem crossing to the lowest energy triplet state occurs in $CsPbBr_3$. The $CsPbBr_3$ displays remarkable light-absorption characteristics and exhibits considerable potential for diverse applications in visible light-catalyzed organic reactions, owing to its inherent efficiency in synthesis and superior yield potential.

Table 2, entry 4 documents the implementation of the chemical reaction under light-deficient conditions, notwithstanding the concurrent utilization of $CsPbBr_3$. The results of the control experiments conducted under controlled conditions reveal that the reaction yield demonstrates a negligible level in the absence of visible light, regardless of the presence or absence of daylight. The present study's experimental results, authenticate that product **4f** corresponds to the specific compound identified as entry 4 in Table 2.

Moreover, the notable redox potential span, prolonged excited state duration, and heightened fluorescence quantum yield evinced by $CsPbBr_3$ are likely to confer benefits concerning its catalytic capabilities.

The photocatalyst $CsPbBr_3$ exhibits extraordinary efficiency in facilitating various organic conversions. In its unexcited ground state, the current photocatalyst displays restricted effectiveness as a unipolar electron-accepting and -donating agent. However, following its exposure to visible light, the aforementioned substance achieves an excited state that serves as a strong agent for the transfer of a single electron.

1. **6. The effect of time on the yield reaction**

Moreover, the inquiry unveiled that the co-existence of $CsPbBr_3$, blue light irradiation, and ethanol is essential for the production of results, thereby confirming them as the principal factors dictating the efficacy outputs in the investigation. Moreover, it has been observed that increasing the duration of the reaction does not manifest any significant effect on the output reaction yield, as evidenced by the data presented in Table S1.

**Table S1.** An optimization table on time for the production of **4f**[a]

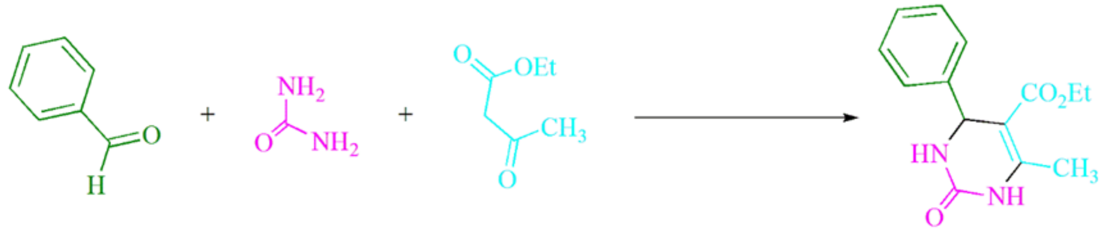


| Entry | Photocatalyst | Solvent (3 mL) | Time (min) | Isolated Yields (%) |
|---|---|---|---|---|
| 1 | $CsPbBr_3$ (1 mol%) | EtOH | 10 | 94 |
| 2 | $CsPbBr_3$ (1 mol%) | EtOH | 15 | 94 |

[a]Reaction conditions: In the experimental methodology, the sample was subjected to 7 W blue LED irradiation at room temperature; The mixture comprised of benzaldehyde (1.0 mmol), ethyl acetoacetate (1.0 mmol), and urea (1.5 mmol).

## 2. Investigating the catalytic activity of a number of photocatalysts

Table S2 displays the comparative catalytic activity of multiple photocatalysts under the same reaction conditions (photocatalyst: 1 mol%, blue LED (7 W), EtOH (3 mL), and room temperature conditions). Based on the findings of the present investigation, $CsPbBr_3$'s elevated yield, coupled

with its superior TON and TOF levels, sets it apart from alternate photocatalysts outlined in Table S2. Furthermore, the capability to retrieve and reuse $CsPbBr_3$ represents a crucial aspect of its utilization for industrial applications of scale. This aspect concurrently renders it a cost-efficient and ecologically beneficial alternative. Furthermore, a greater level of catalytic efficiency can be observed as the yield increases, resulting in an elevated numerical representation of both the Turnover Number (TON) and the Turnover Frequency (TOF). The greater the magnitude of these values, the more proficient the catalytic activity. The study provides evidence that $CsPbBr_3$ exhibits a remarkably high degree of catalytic activity, as demonstrated by its impressive TON (turnover number) of 93 and TOF (turnover frequency) of 18.6 Notably, these values stand in stark contrast to those reported for other catalysts included in Table S2.

**Table S2**. This research endeavor aims to examine the catalytic efficacy of various photocatalysts in the context of their utility for the production of **4f**[a].

| Entry | Photoatalyst (1 mol%) | Conditions | Time/Yield (%) | TON | TOF |
|---|---|---|---|---|---|
| 1 | 4CzPN | Blue LED, EtOH, rt | 5 min/73 | 73 | 14.6 |
| 2 | 2CzPN | Blue LED, EtOH, rt | 5 min/66 | 66 | 13.2 |
| 3 | fluorescein | Blue LED, EtOH, rt | 10 min/78 | 78 | 7.8 |

| | | | | | |
|---|---|---|---|---|---|
| 4 | riboflavin | Blue LED, EtOH, rt | 10 min/76 | 76 | 7.6 |
| 5 | erythrosin B | Blue LED, EtOH, rt | 10 min/65 | 65 | 6.5 |
| 6 | rose bengal | Blue LED, EtOH, rt | 10 min/81 | 81 | 8.1 |
| **7** | **$CsPbBr_3$** | **Blue LED, EtOH, rt** | **5 min/93** | **93** | **18.6** |

[a]The synthetic process incorporates three distinct components, specifically benzaldehyde, ethyl acetoacetate, and urea.

The application of blue light in conjunction with halide perovskite that can be recycled has facilitated progress in the well-known Biginelli reaction, thereby enabling the synthesis of 3,4-dihydropyrimidin-2-(1*H*)-ones/thiones using an environmentally sustainable approach. As per the findings, the technique of gifting displays effectiveness as a unified approach in situations where the response conditions are uncomplicated and efficient. The incorporation of renewable energy sources and recyclable photocatalyst in an ethanol solution offers significant appeal to advocates of sustainable chemistry due to its exceptional speed and yield. The pharmaceutical significance of this compound is emphasized by its scale of production reaching up to 50 mmol, which is measured in multigrams. Furthermore, it is concurrent with the synthesis of drug-active pharmaceutical ingredients applicable in practical scenarios. Furthermore, significant characteristics include a streamlined experimental design, heightened substrate durability, cost-

effectiveness, straightforward coating techniques without the need for strenuous separation procedures, and minimal organic waste production per unit of conversion. The current investigation provides evidence to support the proposition that the spread of crop outputs showcases a significant degree of uniformity, ranging between 86%-94% and averaging at 90.4%. In contrast, the chronological parameters related to the process show signs of rapid kinetics, with a time frame of 4-8 minutes, averaging at 5.8 min. A salient observation pertinent to the discourse at hand underscores that this methodology maintains a diverse range of donating and withdrawing functional groups, which consequently promotes the consistent achievement of elevated yields. The susceptibility of the reaction to rationalization is not altered by the nature of the substituent moieties.

## 3. Single-electron transfer (SET)/photoinduced-electron transfer (PET) pathway

In the primary phase, as illustrated in Figure S4. According to Figure S4; left, the photocatalyst (**PC**) acts as the electrophilic species, enabling the transference of an electron to a suitable electron acceptor (**A**) through a mechanism involving single-electron exchange [1]. The aforementioned occurrence may be explicated in an academic manner as the process of electron transfer facilitated by the (**PC$^*$**) as an appropriate reducing agent, taking place via a reductive mechanism and resulting in electron exchange with a recipient molecule (**A**) through the utilization of a single-electron transfer mechanism. The procedure outlined above instigates the genesis of the anionic radical characterized by a negative charge in the acceptor (**A$^{\bullet-}$**) and the photocatalyst's oxidized state (**PC$^{\bullet+}$**), as documented in [1]. The species in question exhibits oxidizing characteristics and possesses the capability to accept an electron emerging from a donor (**D**), thereby restoring the original state of the photocatalyst and accomplishing the photocatalytic operation [1].

In a reduction-quenching process, as illustrated in Figure S4. In the photochemical reaction of the photosensitizer, Figure S2 (right) [1], the excited state of the photosensitizer, termed (**PC***), serves as an oxidant by receiving an electron from an exogenous electron donor, denoted as (**D**). As a result of this process, the radical cation of the donor, ($\mathbf{D}^{\bullet+}$), and the reduced form of the catalyst, ($\mathbf{PC}^{\bullet-}$) [1], are generated. The restoration of the ground-state photocatalyst is facilitated through electron transfer originating from an acceptor (**A**). From a synthetic standpoint, the aforementioned procedures serve as an exemplary approach for efficiently acquiring exceedingly reactive radical species, wherein visible light constitutes the sole or foremost energy source, and the reaction proceeds under mild operating conditions [1].

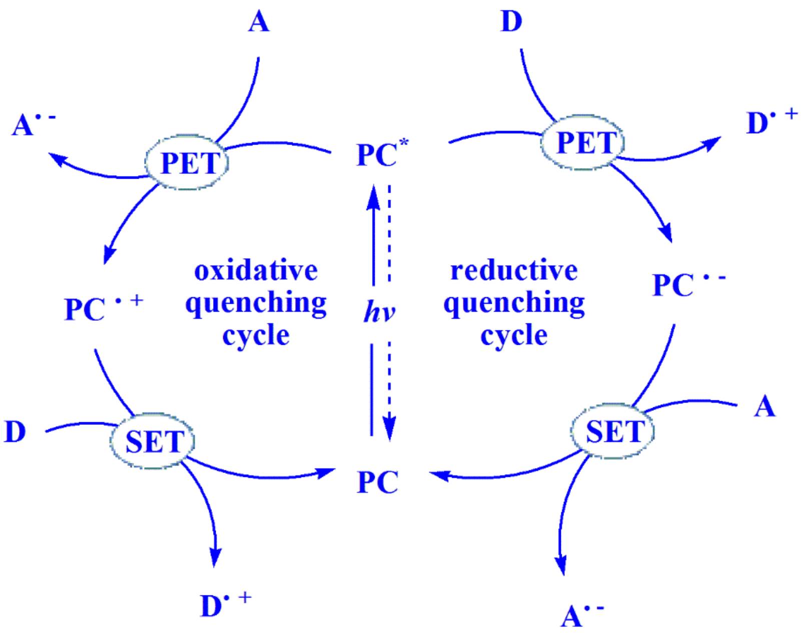


**Figure S4.** This study seeks to examine the essential mechanisms that underlie the oxidative and reductive quenching cycles within photoredox catalytic cycles. Within the domain of electron transfer phenomena, it is commonplace to employ terms that depict the entities involved, such as 'electron donor' or 'electron acceptor', alongside methods of transfer, including 'photoinduced-electron transfer' (PET) and 'single-electron transfer' (SET), among others [1].

## 4. $^{1}$HNMR and $^{13}$CNMR data for compounds (4a, 4b, 4h, 4j, 4k, 4m, 4o, 4p, and 4u)

***5-Ethoxycarbonyl-6-methyl-4-(4-methylphenyl)-3,4-dihydropyrimidin-2(1H)-one (4a)***

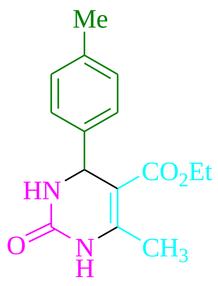


Yield: 92%; M.p. 218-220 °C; $^{1}$HNMR (400 MHz, DMSO-$d_6$): 1.11 (3H, t, *J*= 7.2 Hz, $\underline{CH_3}CH_2$), 2.25 (3H, s, $CH_3$), 2.27 (3H, s, $CH_3$), 3.99 (2H, q, *J*=7.2 Hz, $CH_2O$), 5.11 (1H, s, $H_{benzylic}$), 7.13 (4H, s, $H_{Ar}$), 7.70 and 9.17 (2H, 2s, 2NH); $^{13}$CNMR (100 MHz, DMSO-$d_6$): 14.0 ($\underline{CH_3}$-$CH_2O$), 17.6 ($\underline{CH_3}$-CH=CH), 21.1 ($CH_3$), 53.7 (Ar-CHN), 59.1 ($CH_3$-$\underline{CH_2}O$), 99.2, 123.3 ($CH_3$-$\underline{CH}$=$\underline{CH}$), 126.8, 127.8, 128.2, 137.3, 144.7 and 148.0 ($C_{Ar}$), 151.9 (C=ONH), 165.3 (C=O ester).

***5-Methoxycarbonyl-6-methyl-4-(4-nitrophenyl)-3,4-dihydropyrimidin-2(1H)-one (4b)***

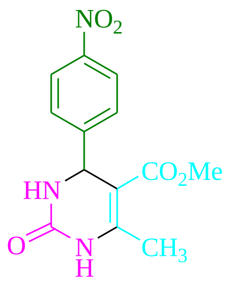

Yield: 91%; M.p. 215-217 °C; $^{1}$HNMR (400 MHz, DMSO-d$_6$): 2.28(3H, s, $CH_3$), 3.55 (3H, s, $OCH_3$), 5.28 (1H, s, $H_{benzylic}$), 7.52 (2H, d, *J*= 8.8Hz, $H_{Ar}$), 8.23 (2H, d, *J*= 8.8Hz, $H_{Ar}$), 7.93 and 9.40 (2H, 2s, 2NH).

***5-Methoxycarbonyl-6-methyl-4-(4-methylphenyl)-3,4-dihydropyrimidin-2(1H)-one (4h)***

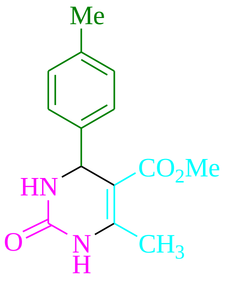


Yield: 94%; M.p. 222-224 °C; $^{1}$HNMR (400 MHz, DMSO-d$_6$): 2.25 (3H, s, $CH_3$), 2.28 (3H, s, $CH_3$), 3.53 (3H, s, $OCH_3$), 5.11 (1H, s, $H_{benzylic}$), 7.01-7.07 (3H, m, $H_{Ar}$), 7.18-7.22 (1H, m, $H_{Ar}$), 7.71 and 9.18 (2H, 2s, 2NH) ppm.

***5-Ethoxycarbonyl-6-methyl-4-(2,4–dimethoxyphenyl)-3,4-dihydropyrimidin-2(1H)-one (4j)***

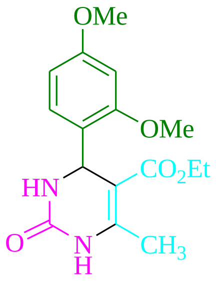


Yield: 91%; M.p. 208-210 °C; $^{1}$HNMR (400 MHz, DMSO-d$_6$): 1.05 (3H , t, *J*= 7.2 Hz, $\underline{CH_3}CH_2$), 2.27 (3H, s, $CH_3$), 3.76 ( 3H, s, $OCH_3$), 3.82 (3H, s, $OCH_3$), 3.93 (2H, q, *J*= 7.2 Hz, $CH_2O$), 5.41

(1H, s, $H_{benzylic}$), 6.45 (1H, dd, *J*=8.4 Hz, J= 2.4 Hz, $H_{Ar}$), 6.54 (1H, d, *J*=2.4 Hz, $H_{Ar}$), 6.94 (1H, d, *J*=8.4 Hz, $H_{Ar}$), 7.21 and 9.08 (2H, 2s, 2NH) ppm; $^{13}$CNMR (100 MHz, DMSO-$d_6$): 14.5 ($\underline{CH_3}$-$CH_2O$), 18.2 ($CH_3$-CH=CH), 48.9 (Ar-CHN), 55.6 and 55.7 ($2OCH_3$), 59.44 ($CH_3$-$\underline{CH_2}$O), 98.9, 104.8 ($CH_3$-CH=CH), 124.6, 124.7,128.2, 148.9, 152.6 and 158.0 ($C_{Ar}$), 160.3 (C=ONH), 165.9 (C=O ester).

***5-Ethoxycarbonyl-6-methyl-4-(4-fluorophenyl)-3,4-dihydropyrimidin-2(1H)–one (4k)***

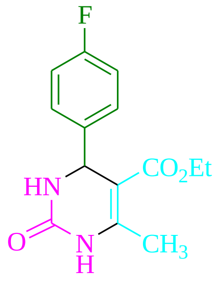


Yield: 92%; M.p. 175-176 °C; $^1$HNMR (300 MHz, DMSO-$d_6$): 1.11 (3H , t, *J*= 9.6 Hz, $\underline{CH_3}CH_2$), 2.25(3H, s, $CH_3$), 3.99 (2H, q, *J*=9.6 Hz, $CH_2O$), 5.14 (1H, s, $H_{benzylic}$), 7.13-7.20 (2H, m, $H_{Ar}$), 7.24-7.29 (2H, m, $H_{Ar}$), 7.78 and 9.25 (2H, 2s, 2NH).

***5-Ethoxycarbonyl-6-methyl-4-(3-methylphenyl)-3,4-dihydropyrimidin-2(1H)–one (4m)***

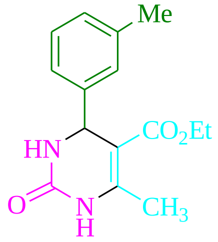

Yield: 90%; M.p. 202-204 °C; $^{1}$HNMR (400 MHz, DMSO-$d_6$): 1.11 (3H , t, *J*= 7.2 Hz, $\underline{CH_3}CH_2$), 2.24 (3H, s, $CH_3$), 2.28 (3H, s, $CH_3$), 3.99 (2H, q, *J*=7.2 Hz, $CH_2O$), 5.11 (1H, s, $H_{benzylic}$), 7.02-7.06 (3H, m, $H_{Ar}$), 7.18-7.22 (1H, m, $H_{Ar}$), 7.69 and 9.15 (2H, 2s, 2NH).

***5-Ethoxycarbonyl-6-methyl-4-(2-chlorophenyl)-3,4-dihydropyrimidin-2(1H)–one (4o)***

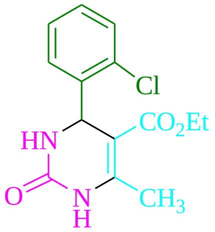


Yield: 88%; M.p. 222-224 °C; $^{1}$HNMR (400 MHz, DMSO-$d_6$): 1.00 (3H , t, *J*= 9.2 Hz, $\underline{CH_3}CH_2$), 2.31 (3H, s, $CH_3$), 4.02 (2H, q, *J*=9.2 Hz, $CH_2O$), 5.63 (1H, s, $H_{benzylic}$), 7.25-7.34 (3H, m, $H_{Ar}$), 7.41 (1H, d, *J*=8.8 Hz, $H_{Ar}$), 7.73 and 9.29 (2H, 2s, 2NH).

***5-Ethoxycarbonyl-6-methyl-4-(3,4,5–trimethoxyphenyl)-3,4-dihydropyrimidin-2(1H)-thione (4p)***

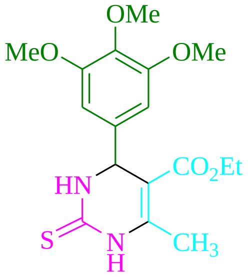

Yield: 86%; M.p. 196-198 °C; $^{1}$HNMR (400 MHz, DMSO-d$_6$): 1.16 (3H , t, *J*= 7.2 Hz, <u>CH$_3$</u>CH$_2$), 2.30 (3H, s, CH$_3$), 3.65 (3H, s, OCH$_3$), 3.74 (6H, s, 2OCH$_3$), 4.07 (2H, q, *J*=7.2 Hz, CH$_2$O), 5.16 ( 1H, s, H$_{benzylic}$), 6.52 ( 2H, s, H$_{Ar}$), 9.65 and 10.37 (2H, 2s, 2NH).

***5-Ethoxycarbonyl-6-methyl-4-phenyl-3,4-dihydropyrimidin-2(1H)-thione (4u)***

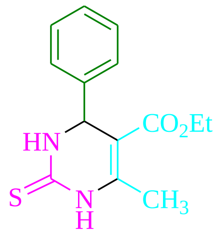


Yield: 92%; M.p. 209-211 °C; $^{1}$HNMR (400 MHz, DMSO-d$_6$): 1.11 (3H , t, *J*= 7.2 Hz, <u>CH$_3$</u>CH$_2$), 2.31 (3H, s, CH$_3$), 4.02 (2H, q, *J*=7.2 Hz, CH$_2$O), 5.19 (1H, s, H$_{benzylic}$), 7.23 (2H, d, *J*=7.2 Hz, H$_{Ar}$), 7.28 (1H, t, *J*=7.2 Hz, H$_{Ar}$), 7.36 (2H, t, *J*=7.2 Hz, H$_{Ar}$), 9.68 and 10.36 ( 2H, 2s, 2NH).

## 5. $^{1}$HNMR and $^{13}$CNMR files for compounds (4a, 4b, 4h, 4j, 4k, 4m, 4o, 4p, and 4u)

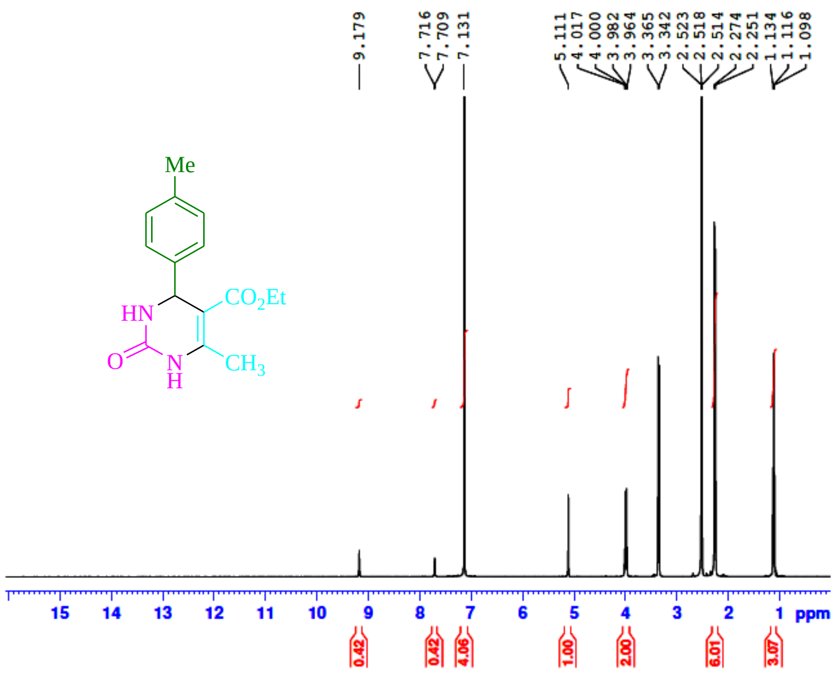


**Figure S5.** $^{1}$HNMR spectrum of compound (400 MHz, DMSO-$d_6$) of **4a**

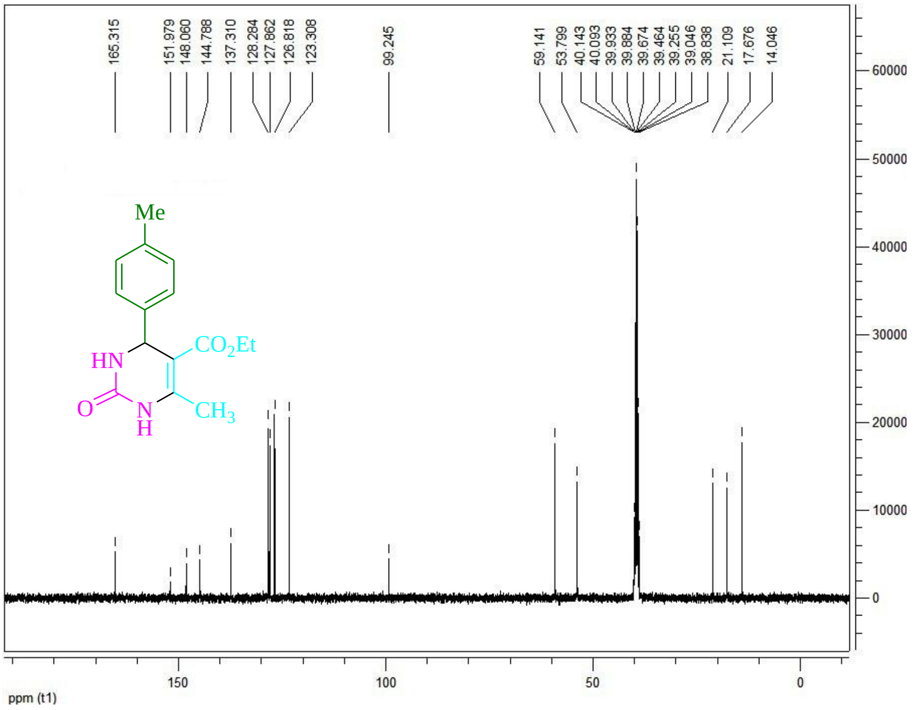


**Figure S6.** $^{13}$CNMR spectrum of compound (100 MHz, DMSO-d$_6$) of **4a**

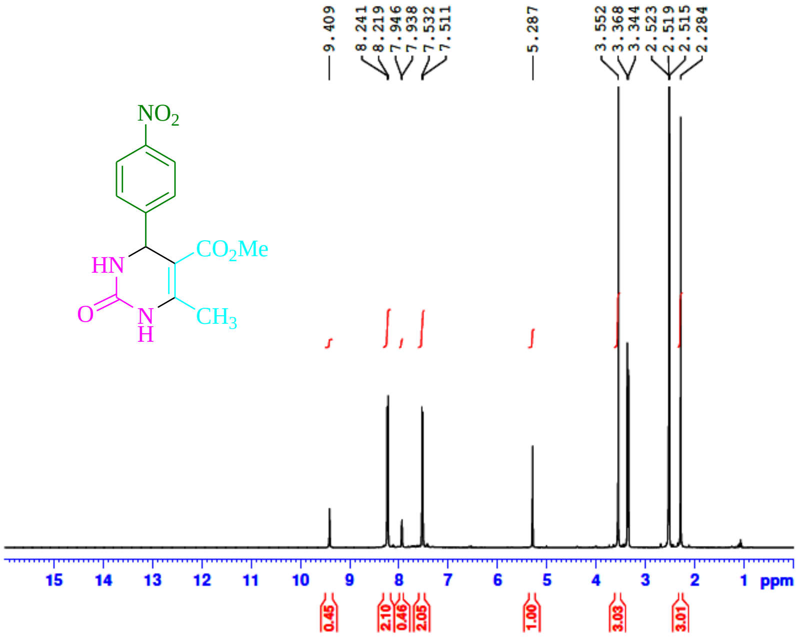


**Figure S7.** $^{1}$HNMR spectrum of compound (400 MHz, DMSO-d$_6$) of **4b**

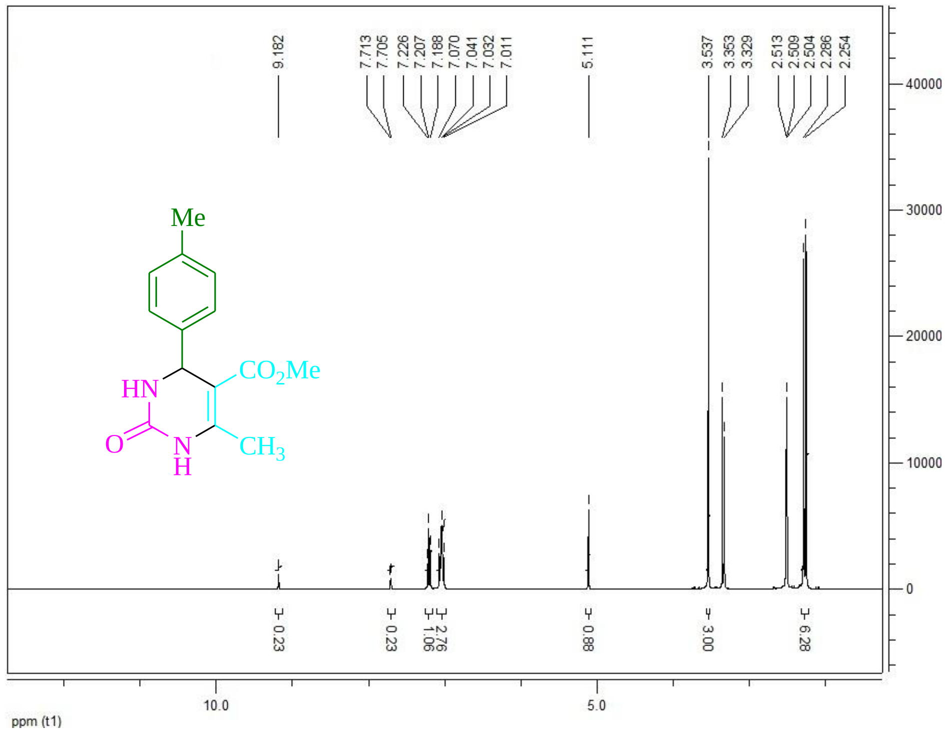

**Figure S8**. $^{1}$HNMR spectrum of compound (400 MHz, DMSO-d$_6$) of **4h**

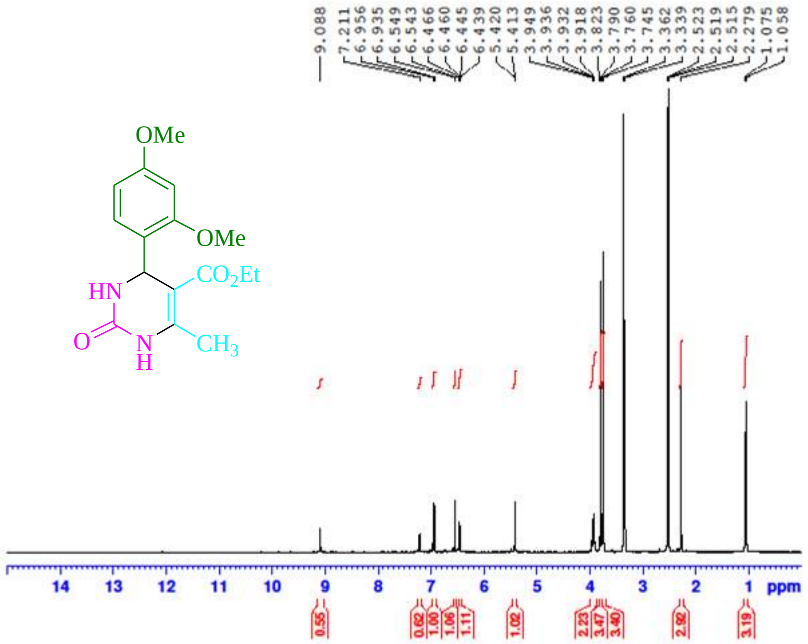

**Figure S9.** $^{1}$HNMR spectrum of compound (400 MHz, DMSO-d$_6$) of **4j**

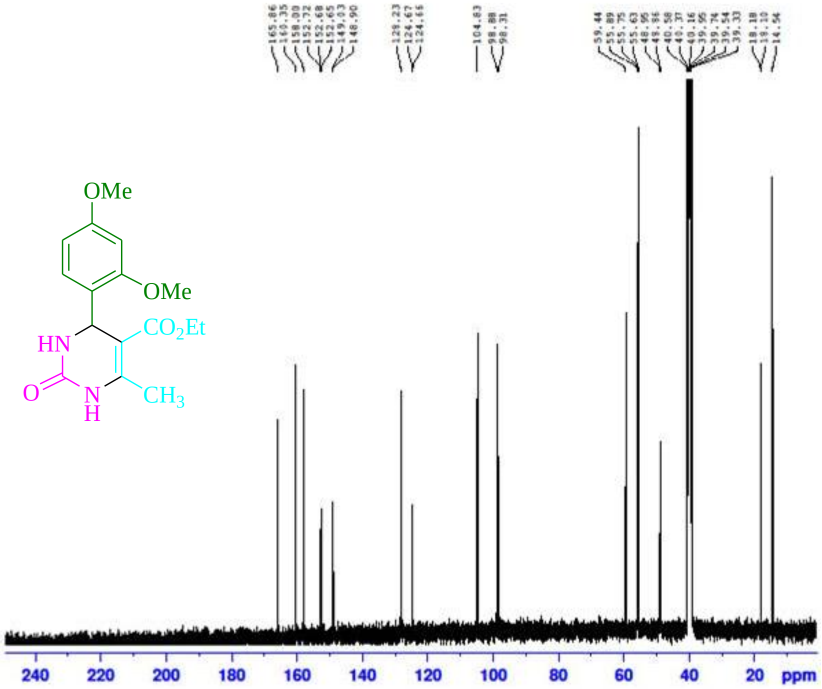

**Figure S10.** $^{13}$CNMR spectrum of compound (100 MHz, DMSO-d$_6$) of **4j**

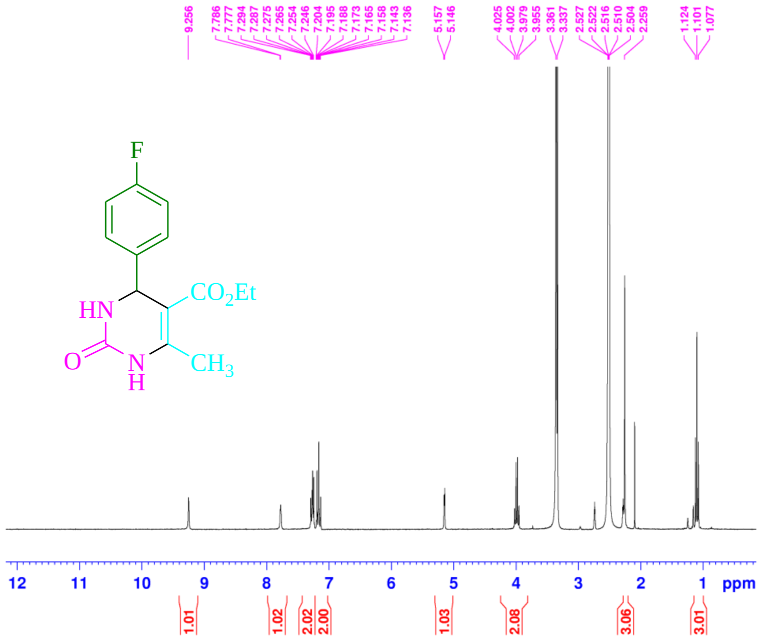

**Figure S11.** $^{1}$HNMR spectrum of compound (300 MHz, DMSO-d$_6$) of **4k**

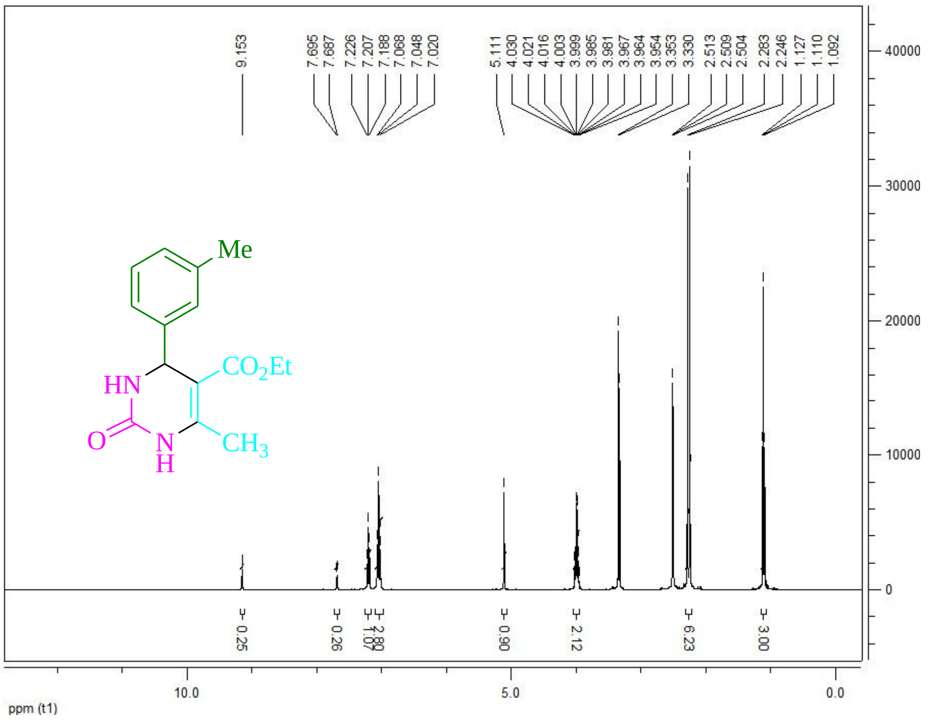

**Figure S12**.$^{1}$HNMR spectrum of compound (400 MHz, DMSO-d6) of **4m**

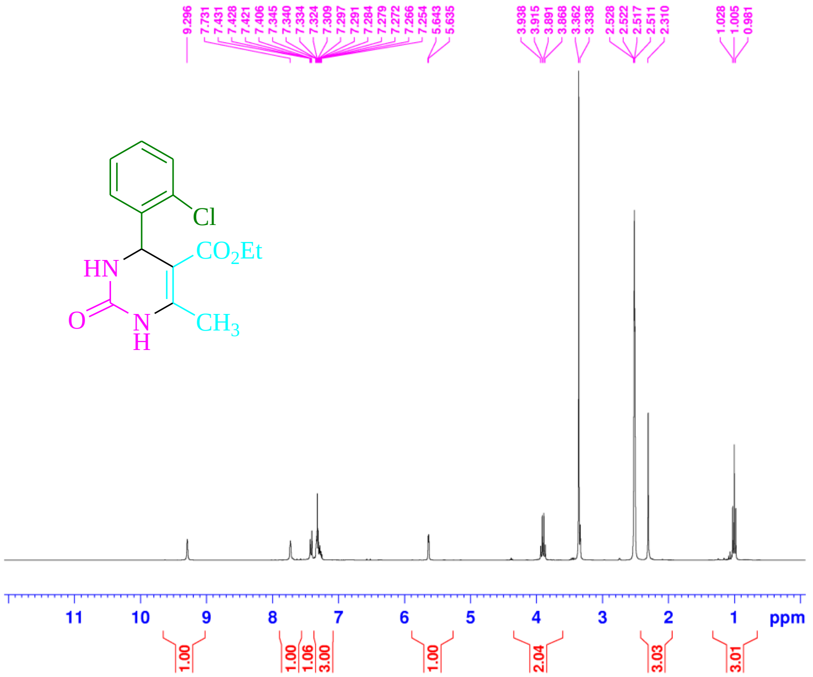

**Figure S13.** $^{1}$HNMR spectrum of compound (400 MHz, DMSO-d6) of **4o**

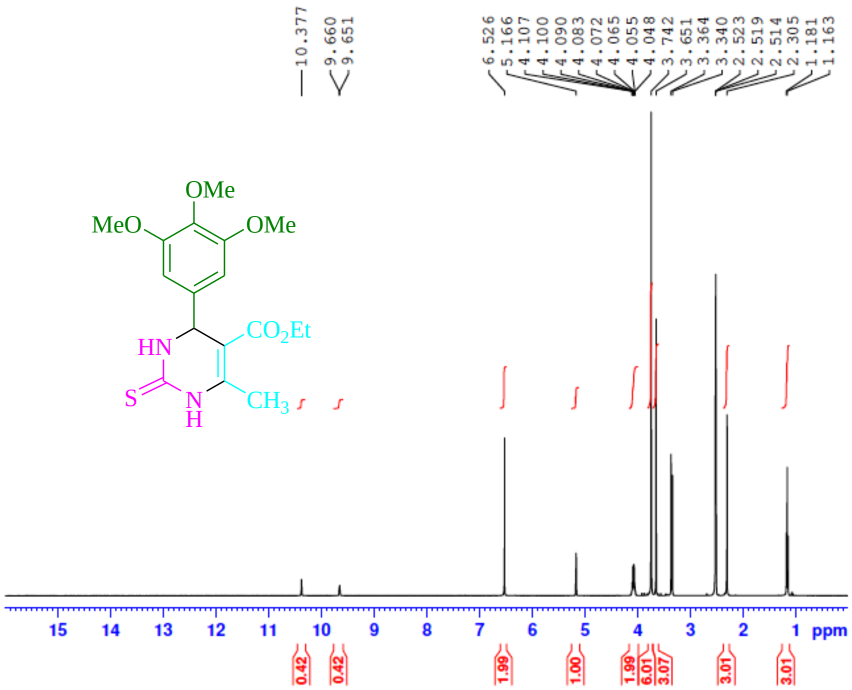


**Figure S14.** $^{1}$HNMR spectrum of compound (400 MHz, DMSO-d$_6$) of **4p**

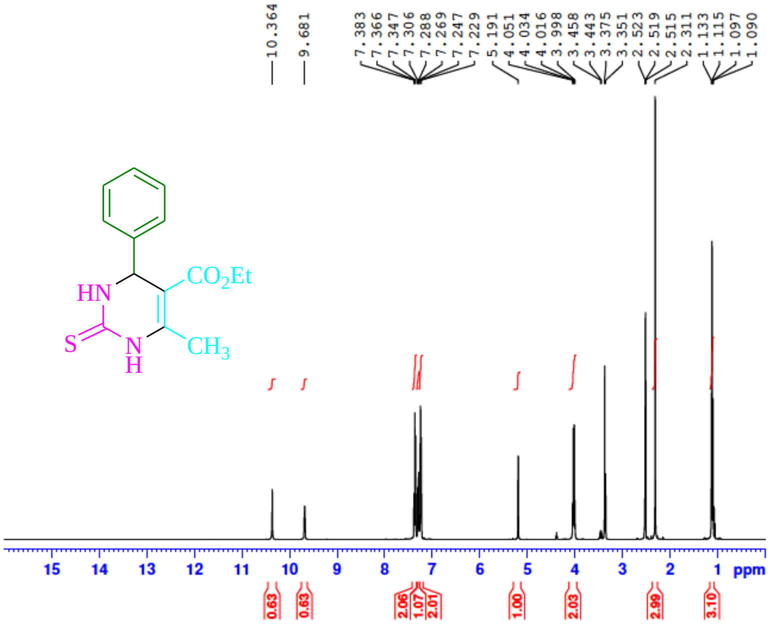


**Figure S15.** $^{1}$HNMR spectrum of compound (400 MHz, DMSO-d$_6$) of **4u**

## 6. References

[1] A. D. Kinghorn, H. Falk, S. Gibbons, Y. Asakawa, J. K. Liu, V. M. Dirsch, editors. Modern Photocatalytic Strategies in Natural Product Synthesis, Springer Nature (2023).
